# Impact of neighborhood-level COVID-19 mortality on the increase in drug overdose mortality in New York City during the COVID-19 pandemic

**DOI:** 10.1186/s40621-024-00548-8

**Published:** 2024-11-22

**Authors:** Wuraola Olawole, Guohua Li, Ziqi Zhou, Zhixing Wu, Qixuan Chen

**Affiliations:** 1 Department of Biostatistics, Columbia University Mailman School of Public Health, New York, NY 10032 USA; 2 School of Nursing, Johns Hopkins University, Baltimore, MD 21205 USA; 3 Department of Anesthesiology, Columbia University Vagelos College of Physicians and Surgeons, New York, NY 10032 USA; 4 Department of Epidemiology, Columbia University Mailman School of Public Health, New York, NY 10032 USA; 5 Center for Research on Cultural and Structural Equity, Nathan S. Kline Institute for Psychiatric Research, Orangeburg, NY 10962 USA

**Keywords:** COVID-19 death rate, Drug overdose mortality, Neighborhood characteristics, UHF neighborhoods

## Abstract

**Background:**

Overdose mortality increased substantially during the COVID-19 pandemic, but it is unclear to what extent the COVID-19 mortality had contributed to this increase at the neighborhood level.

**Methods:**

This was an ecological study based on New York City United Hospital Fund (NYC UHF) neighborhood-level data from 2019 to 2021, split into two time-windows: pre-COVID (2019) and during-COVID (2020 and 2021). Linear regression models were used to estimate the effect of cumulative COVID-19 mortality on the increase in drug overdose mortality from the pre-COVD to during-COVID periods at the neighborhood level, with and without adjusting for neighborhood characteristics.

**Results:**

Drug overdose mortality rate increased from 21.3 to 33.4 deaths per 100,000 person-years across NYC UHF neighborhoods from pre-COVID to during-COVID. For each additional COVID-19 death per 1,000 person-years at the neighborhood level, the increase in drug overdose mortality rose 2.4 (95% CI: 1.7, 3.3) times. Furthermore, neighborhoods with a higher percentage of Hispanic residents, a higher percentage of single-person households, and a higher percentage of residents with health insurance experienced significantly larger increases in drug overdose mortality. In contrast, neighborhoods with a higher percentage of residents aged 75 and older had a smaller increase in drug overdose mortality.

**Conclusions:**

NYC neighborhoods with higher cumulative COVID-19 mortality experienced a greater increase in drug overdose mortality during the first two years of the COVID-19 pandemic.

## Background

Deaths from drug overdose have been on the rise in the United States since the late 1990s through 2020 (Hedegaard et al. 2021), with a dramatic surge in recent years (Spencer et al. 2024). Nationwide, overdose deaths increased by about 30% from 2019 to 2020 and almost doubled by 2021 (Hedegaard et al. 2021; CDC 2022). This continuing increase represents a serious public health challenge and an economic burden to the nation (Florence et al. 2020; Luo et al. 2021). According to the National Center for Health Statistics (NCHS), drug overdose mortality from 1999 to 2020 has significantly contributed to the increase in national death rates and the reduction in life expectancy (Spencer et al. 2022; Mattson et al. 2021).

One major population center impacted by this rising overdose epidemic is New York City (NYC) (Holshue et al. 2020). Despite targeted efforts to address the crisis (Chen et al. 2020), NYC neighborhoods continue to experience significant disparities in drug overdose mortality across different boroughs (Han et al. 2019; Cano & Gelpí-Acosta 2021). Compounding this issue, NYC was also an early epicenter of the COVID-19 pandemic in the United States, suffering high case and death counts that varied widely across neighborhoods (Wadhera et al. 2020; Thompson et al. 2020; Zhong et al. 2022).

Between 2020 and 2021, NYC faced both the ongoing COVID-19 pandemic and a worsening drug overdose epidemic, both of which disproportionally affected neighborhoods across the city (CDC 2020a). Overdose death rates in 2020 and 2021 (during-COVID) reached their highest levels since reporting commenced in 2000 (Nolan et al. 2020, 2021; Askari et al. 2023). Thus, while the COVID-19 pandemic was disrupting lives across the city, the drug overdose crisis escalated, creating a dual public health emergency that exposed and exacerbated neighborhood-level disparities.

The COVID-19 pandemic may have exacerbated drug overdose mortality at the neighborhood level. Neighborhood-level drug overdose mortality and COVID-19 mortality may share some common risk factors. This is the syndemic concept where the diseases co-occur due to the fact that they share risk factors (Mendenhall et al. 2022). Furthermore, individuals experiencing loss due to COVID-19 may turn to drugs to cope with depression and traumatic grief. This is especially plausible given the limited social interactions, restricted access to grief support, and reduced health service during the pandemic (Hosseinzadeh et al. 2022). Finally, economic hardships brought on by COVID-19 outcomes, particularly deaths, may have further exacerbated drug overdose in communities already facing socioeconomic inequality (Roman et al. 2022; Mutikani 2020).

Although the neighborhood disparities and socio-economic risk factors for drug overdose mortality and COVID-19 death rate have been previously studied individually (Altekruse et al. 2020; Zhong et al. 2022), much less is known about the association between the COVID-19 mortality and the increase in drug overdose mortality during the COVID-19 pandemic in NYC at neighborhood level. The objective of this study was to assess if neighborhoods with higher COVID-19 mortality rates experienced a greater increase in drug overdose mortality during the COVID-19 pandemic compared to the pre-COVID period in NYC.

## Methods

### Study design and data source

This was an ecological study based on neighborhood-level data from NYC. Data on COVID-19 mortality and drug overdose mortality at the United Hospital Fund (UHF) neighborhood level were acquired from the NYC DOHMH (New York City Department of Health and Mental Hygiene 2022; New York City Department of Health and Mental Hygiene 2023a). There are 42 UHF neighborhoods in NYC, and the boundaries are based on zip code tabulation areas, designed for health research, and designated to approximate New York City Community Districts (New York City Department of Health and Mental Hygiene 2023b). The NYC neighborhood-level demographics and socio-economic data, organized by zip codes, were obtained from the US Census Bureau using the 2020 American Community Survey (ACS) 5-year estimates (U.S. Census Bureau 2022). To reconcile the outcome data with the covariate data, we used a mapping resource provided by the NYC DOHMH to convert zip codes to UHF neighborhoods.

### Outcome measure

The outcome measure was the total count of the number of drug overdose deaths in each UHF neighborhood from 2019 to 2021. We split the study period into two time-windows: pre-COVID and during-COVID, where the 2019 drug overdose data were collected and used as the pre-COVID measure, and the 2020 and 2021 data were used as the during-COVID measures. The population size in Census 2020 in each UHF was used as the denominator in calculating the drug overdose mortality rates. The change in drug overdose mortality rate was calculated by subtracting the 2019 mortality rate from the mortality rate in 2020–2021.

### Key predictor

We focused on COVID-19 mortality as the key predictor which was calculated as the cumulative COVID-19 related death per 1,000 person-years from March 2020 to December 2021 for each UHF neighborhood as reported by NYC DOHMH.

### Neighborhood characteristics

We examined several neighborhood characteristics, including borough (Manhattan, Bronx, Brooklyn, Queens, Staten Island), percentage of age group (≥ 19, 20–39, 40–59, 60–74, and ≥ 75), percentage of male residents, percentage of race/ethnicity (Asian, Black, Hispanic), percentage of households with various sizes (1, 2, 3, ≥ 4 members), percentage with high school diploma, percentage with health insurance, and median household income.

### Statistical analysis

We used frequencies and means with standard deviations (SDs) to summarize the outcome, key predictor, and neighborhood characteristics. We generated maps of drug overdose mortality rate by UHF neighborhoods in each year from 2019 to 2021. We also created a scatter plot of the cumulative COVID-19 death rates and change in drug overdose mortality rate from pre-COVID to during-COVID by UHF neighborhoods. We fit linear regression models to assess the associations of the COVID-19 death rates and neighborhood characteristics with the increase in drug overdose mortality rate during the COVID-19 pandemic. To address the right skewness in the data, we applied a natural logarithm transformation to the increase in drug overdose mortality. We started with marginal models that include one covariate at a time, followed by the multivariable model that selected covariates using stepwise model selection by Akaike Information criteria (AIC). We used R version 4.3.0 for all the analyses in this study.

## Results

### Demographic and socioeconomic characteristics

Forty-two UHF neighborhoods were included in the study, including 10 in Manhattan, 11 in Brooklyn, 10 in Queens, 7 in Bronx, and 4 in Staten Island. Table 1 shows that neighborhoods in Manhattan had the highest percentage of residents aged 20–39 (37.8%) and the highest percentage of residents 75 years and older (7.3%), while neighborhoods in Bronx had the highest percentage of residents younger than 20 years of age (27.3%). The percentage of male residents across all boroughs were similar. Neighborhoods in Bronx had the highest percentage of Black (28.6%) and Hispanic (55.4%) population; Staten Island neighborhoods had the highest percentage of White population (54.3%); and neighborhoods in Queens had the highest percentage of Asian population (25.6%). Neighborhoods in Manhattan had the highest percentage of single member households (45.8%) while Staten Island neighborhoods had the highest percentage of households with 4 or more members (32.2%). The health insurance coverage was high across all boroughs (93.5%). The average median household income for Manhattan neighborhoods was the highest at $104,000, followed by Staten Island at $84,000, Queens at $79,000, and Brooklyn at $68,000. Bronx had the least median household income at $48,000.

**Table 1:** Mean (standard deviation) of sociodemographic characteristics in NYC based on the 42 United Hospital fund (UHF) neighborhoods, using the 2020 US Census Bureau American Community Survey (ACS) 5-year estimates

	Bronx (*n* = 7)	Brooklyn (*n* = 11)	Manhattan (*n* = 10)	Queens (*n* = 10	Staten Island (*n* = 4)	Total (*n* = 42)
COVID death rate per 1000 person-years	2.46 (0.35)	2.06 (0.61)	1.35 (0.65)	2.22 (0.58)	2.12 (0.53)	2.00 (0.67)
Percentage of residents by age in years						
0–19	27.3 (3.7)	24.6 (3.2)	16.1 (4.0)	22.2 (3.4)	24.6 (2.2)	22.5 (5.1)
20–39	29.2 (2.8)	32.5 (6.8)	37.8 (4.0)	28.7 (5.8)	25.8 (1.5)	31.7 (6.3)
40–59	24.5 (0.8)	23.9 (2.4)	24.7 (1.9)	26.7 (1.9)	27.0 (0.7)	25.2 (2.1)
60–74	12.8 (3.0)	13.1 (3.0)	14.1 (2.4)	15.3 (2.2)	16.1 (1.5)	14.1 (2.7)
≥ 75	6.1 (2.8)	5.9 (2.0)	7.3 (2.0)	7.0 (1.4)	6.6 (1.4)	6.6 (2.0)
Percentages of Males	46.8 (1.2)	47.6 (2.0)	47.6 (2.4)	48.4 (1.3)	48.5 (0.6)	47.8 (1.8)
Percentages of Race/Ethnicity						
White	10.4 (12.5)	35.6 (22.0)	49.0 (23.1)	27.3 (14.8)	54.3 (20.9)	34.4 (23.2)
Black	28.6 (15.2)	27.1 (27.1)	12.3 (14.7)	17.4 (20.9)	11.7 (11.4)	20.0 (20.4)
Hispanic	55.4 (14.8)	21.1 (12.7)	21.9 (17.5)	25.1 (10.3)	21.0 (10.7)	28.0 (18.1)
Asian	3.3 (2.8)	12.7 (11.6)	13.4 (6.9)	25.6 (12.9)	10.7 (5.5)	14.2 (11.7)
Others	0.8 (0.3)	0.5 (0.2)	0.6 (0.3)	1.7 (2.0)	0.2 (0.1)	0.8 (1.1)
Percentage of household (HH) by size						
1	31.7 (3.8)	28.1 (4.3)	45.8 (6.5)	25.4 (5.0)	23.0 (3.5)	31.8 (9.5)
2	24.6 (2.4)	29.3 (4.8)	31.1 (2.3)	28.2 (3.7)	27.2 (1.4)	28.5 (3.9)
3	18.5 (1.7)	17.3 (1.7)	12.0 (3.0)	18.3 (1.5)	17.7 (0.8)	16.5 (3.2)
≥ 4	25.3 (4.0)	25.3 (7.0)	11.1 (4.5)	28.1 (7.1)	32.2 (3.1)	23.2 (9.0)
Percentage of residents with health insurance	92.3 (1.9)	93.0 (2.6)	95.6 (2.1)	91.9 (2.8)	95.5 (1.6)	93.5 (2.7)
Percentage of residents with high school diploma or higher	74.7 (6.6)	82.6 (7.8)	89.0 (8.0)	84.3 (5.1)	87.7 (3.9)	83.7 (8.0)
Median annual household income ($10,000)	4.8 (1.9)	6.8 (2.0)	10.4 (4.8)	7.9 (1.1)	8.4 (1.3)	7.7 (3.2)

### Spatial representation of drug overdose and COVID-19 mortality rates

**Fig. 1:**
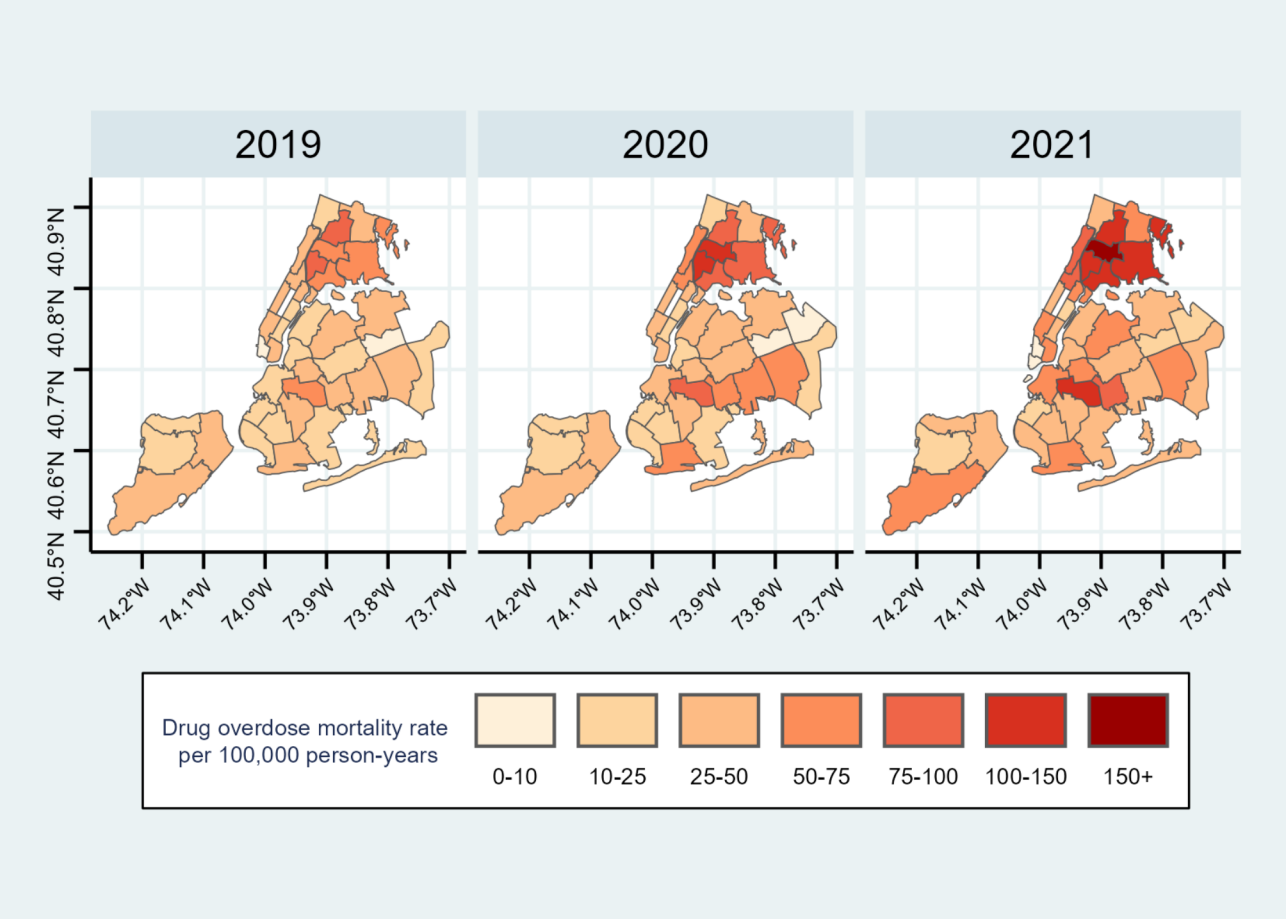
Geographic distribution of drug overdose mortality rate per 100,000 person-years in the 42 NYC UHF neighborhoods from 2019 to 2021

Figure 1 shows the geographic distribution of the drug overdose mortality rate across the 42 NYC UHF neighborhoods from 2019 to 2021. Darker colors indicate higher mortality rates in a given neighborhood. Disparities in the geographic distribution of drug overdose mortality rates were observed over these three years. Neighborhoods in Bronx consistently had the highest drug overdose mortality rates, with 37 deaths per 100,000 person-years in 2019. This rate increased during the COVID-19 pandemic to 52 deaths per 100,000 person-years in 2020 and 72 deaths per 100,000 person-years in 2021. Conversely, Queens had the lowest drug overdose mortality rate among the five boroughs, with 14 deaths per 100,000 person-years in 2019, 21 deaths per 100,000 person-years in 2020, and 23 deaths per 100,000 person-years in 2021. Overall, the drug overdose mortality rate rose across all 5 boroughs from 2019 to 2021, as indicated by the progressively darker colors on the maps.

**Fig. 2:**
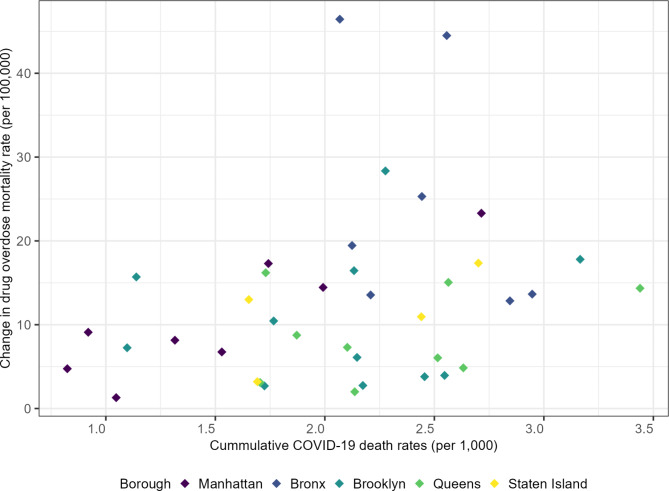
A scatter plot of the change in drug overdose mortality rate from pre-COVID (2019) to during-COVID (2020–2021) against the cumulative COVID-19 death rate (2020–2021) across 42 NYC UHF neighborhoods

Figure 2 shows a scatter plot of the change in drug overdose mortality rate from pre-COVID (2019) to during-COVID (2020–2021) against the cumulative COVID-19 death rate in 2020–2021. There was a moderate positive association between the change in drug overdose mortality rate and the cumulative COVID-19 death rate at UHF neighborhoods.

### Marginal analyses

The first column of Table 2 shows the results of the marginal analyses of the linear regression models. Neighborhoods with higher cumulative COVID-19 death rates also had a higher drug overdose mortality rate. For each additional COVID-19-related death per 1,000 person-years, the increase in drug overdose mortality rate from 2019 to 2020–2021 rose $\:{e}^{0.457}$ = 1.6 (95% CI: 1.03, 2.4) times. Compared to Manhattan, residents of Bronx experienced a greater increase in drug overdose mortality rates. Neighborhoods with a higher percentage of residents aged 60–74 and those aged 75 and older saw a smaller increase in drug overdose mortality rate, while those with a higher percentage of residents under 20 years experienced a larger increase. Additionally, neighborhoods with a higher percentage of Black or Hispanic residents had a greater rise in overdose mortality rates, while those with a higher percentage of Asian residents saw a smaller increase. Finally, neighborhoods with higher percentage of residents holding a high school diploma and higher median annual household income experienced a smaller increase in drug overdose mortality rates.

**Table 2:** Estimated effects and 95% confidence intervals (CIs) of COVID-19 death rate and other covariates on the natural log-transformed increase in drug overdose mortality from 2019 to 2020–2021 among the 42 UHFs in NYC using marginal and multivariable linear regression models. The marginal models regressed on one covariate at a time. The covariates in the multivariable model were selected using stepwise selection by AIC

Covariates	Marginal Model Regression coefficient (95% CI)	Multivariable Model Regression coefficient (95% CI)
COVID-19 death rate in 1,000 person-years	**0.457 (0.033**,** 0.880)**	**0.857 (0.512**,** 1.203)**
Borough		
Manhattan	Ref	
Bronx	**0.998 (0.188**,** 1.809)**	
Brooklyn	– 0.031 (– 0.759, 0.696)	
Queens	– 0.142 (– 0.903, 0.619)	
Staten Island	0.155 (– 0.804, 1.115)	
Age in years		
10% increase among age ≤ 19	**0.054 (0.001**,** 0.107)**	
10% increase among age 20–39	0.012 (– 0.035, 0.059)	
10% increase among age 40–59	– 0.058 (– 0.191, 0.076)	
10% increase among age 60–74	**– 0.133 (– 0.232**,** – 0.033)**	
10% increase among age ≥ 75	**– 0.172 (– 0.302**,** – 0.041)**	**– 0.221** (– **0.333**,** – 0.109**)
Sex		
10% increase in Male population	– 0.069 (– 0.219, 0.082)	
Race/Ethnicity		
10% increase in Black population	**0.013 (0.001**,** 0.026)**	
10% increase in Asian population	**– 0.042 (– 0.063**,** – 0.021)**	
10% increase in Hispanic population	**0.024 (0.011**,** 0.038)**	**0.022** (**0.008**,** 0.035**)
Household (HH) size		
10% increase in 1 member HHs	0.013 (– 0.017, 0.042)	**0.036** (**0.011**,** 0.061**)
10% increase in 2–3 member HHs	– 0.025 (– 0.11, 0.06)	0.045 (– 0.014, 0.104)
10% increase in 4 or more member HHs	– 0.01 (– 0.042, 0.021)	
Health Insurance		
10% increase with health insurance	– 0.018 (– 0.12, 0.084)	**0.146** (**0.056**,** 0.236**)
Education		
10% increase with high school diploma	**– 0.036 (– 0.069**,** – 0.003)**	
Household income		
$10,000 increase in median annual HH income	**– 0.163 (– 0.253**,** – 0.073)**	

Significant findings are highlighted in bold for emphasis

### Multivariable analyses

The second column of Table 2 shows the results of the multivariable analyses. The stepwise model selection by AIC selected COVID-19 death rate and 4 neighborhood characteristics. After adjusting for these neighborhood characteristics at the UHF level, the COVID-19 death rate was positively associated with the increase in the drug overdose mortality rates. For each additional COVID-19-related death per 1,000 person-years, the increase in drug overdose mortality rate rose $\:{e}^{0.856}$= 2.4 (95% CI: 1.7, 3.3) times. Furthermore, neighborhoods with a higher percentage of Hispanic residents, a higher percentage of single-person households, and a higher percentage of residents with health insurance experienced a large increase in drug overdose mortality. In contrast, neighborhoods with a higher percentage of residents aged 75 and older had a smaller increase in overdose mortality rates. The multivariable linear model achieved an R-square of 68.1%, with a partial R-square of 44.4% attributed to the COVID-19 death rate.

**Fig. 3:**
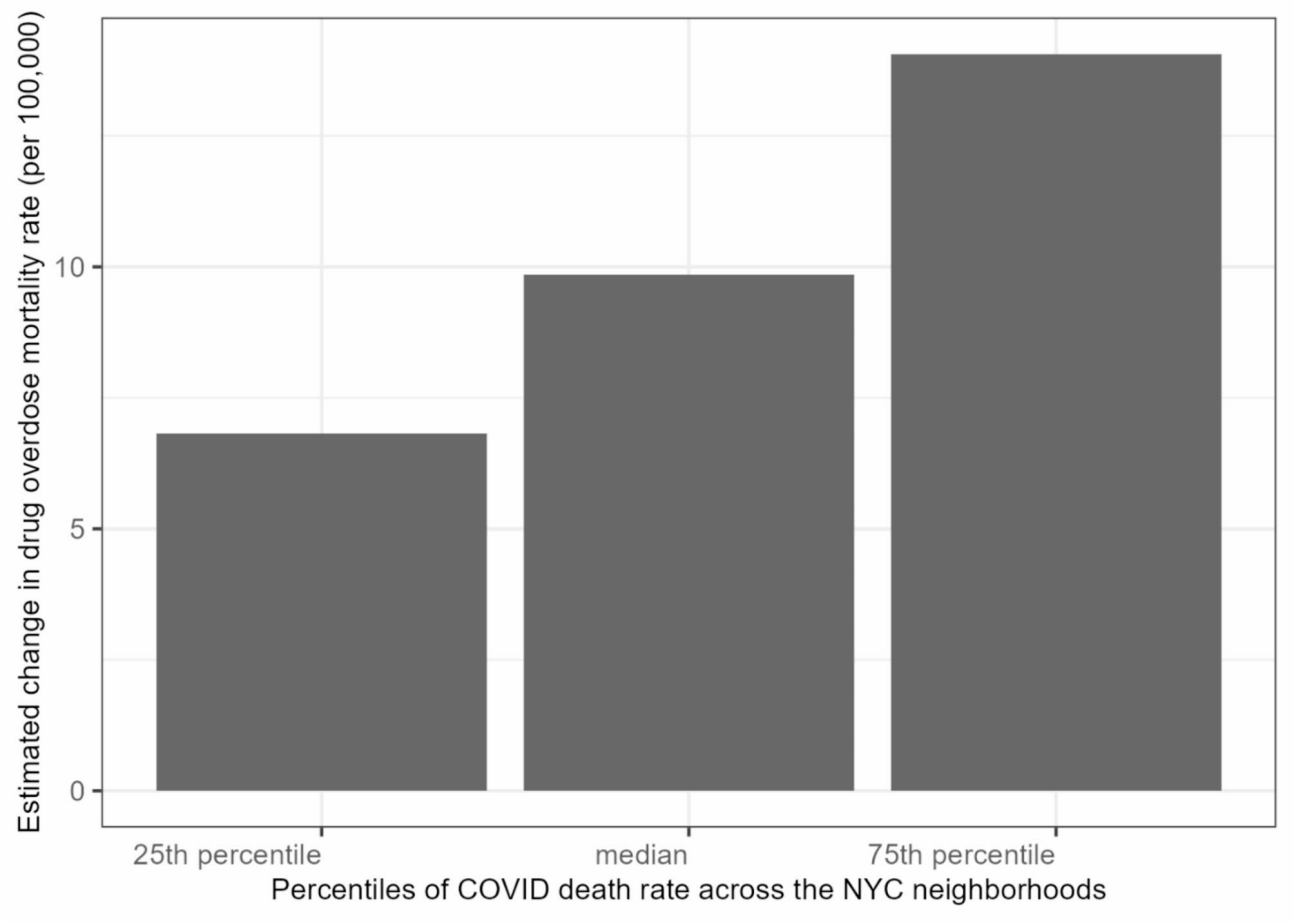
The estimated change in drug overdose mortality rates from pre-COVID (2019) to during-COVID (2020–2021) based on the multivariable model when neighborhood COVID-19 death rates were at their 25th percentile (1.7 per 1000 person-years), median (2.1 per 1000), and 75th percentile (2.5 per 1000), while holding all other covariates in the multivariable model at their average values across the 42 NYC UHF neighborhoods

Figure 3 shows a comparison of estimated increase in drug overdose death rates from pre- to during-COVID based on the multivariable model when the neighborhood-level COVID-19 death rates were at 25th percentile (1.7 COVID-19 related deaths per 1,000 person-years), median (2.1 COVID-19 related deaths), and 75th percentile (2.5 COVID-19 related deaths), while holding all other covariates in the multivariable model at their average values across the 42 UHF neighborhoods. The plot showed that the estimated overdose mortality rate doubled, rising from 6.8 to 14.1 per 100,000 person-years, as the COVID-19 death rate increased from their 25th to 75th percentile.

## Discussion

Using the NYC drug overdose deaths data from 2019 to 2021 and the COVID-19 death rate data from 2020 to 2021, our analyses showed that UHF neighborhoods with higher COVID-19 death rates also had a larger increase in drug overdose mortality rates from the pre-COVID to during-COVID periods. The findings from these analyses also identified vulnerable populations (at UHF level) in NYC at increased risk for drug overdose mortality.

Previous studies have explored the relation between the COVID-19 pandemic and drug overdose. Many focused on access to overdose prevention programs (Alexander et al. 2020; Haley & Saitz 2020; Niles et al. 2020; Ignaszewski 2021), drug overdose mortality trends and disparity (Friedman & Hansen 2022), and the clinical impact of COVID-19 outcomes on patients with substance use disorder (Wang et al. 2020; Vallecillo et al. 2020). These studies found higher risks of severe COVID-19 outcomes and comorbidities in individuals with substance use disorders, but primarily viewed substance use disorder as a risk factor for COVID-19 severity. Few studies have examined drug overdose as the primary outcome, particularly at a population level (Lee & Singh 2023; CDC 2020b), and even fewer have focused on the association between COVID-19 and drug overdose mortality in NYC (Allen et al. 2020).

Our study aimed to assess whether neighborhoods with higher COVID-19 death rates experienced a greater increase in drug overdose mortality during the COVID-19 pandemic compared to the pre-COVID period in NYC. We found that the drug overdose mortality rate increased during the COVID-19 pandemic (2020–2021) compared to the pre-COVID period (2019) in all NYC UHF neighborhoods. By examining the COVID-19 death rate as a key predictor, we found that neighborhoods with higher cumulative COVID-19 deaths in 2020–2021 experienced a greater increase in drug overdose mortality rate during the pandemic. These results suggest that NYC UHF neighborhoods with higher cumulative COVID-19 death rates faced a greater risk of drug overdose mortality during the pandemic compared to pre-COVID.

In NYC and other similar cities, studies (Zhong et al. 2022; Khatri et al. 2021; Altekruse et al. 2020) have shown that certain socio-economic factors that increase a community’s vulnerability to COVID-19 also contribute to a higher risk of drug overdose fatalities. This leads to the syndemic concept where the diseases may co-occur due to the fact that they share risk factors (Mendenhall et al. 2022). This could be one explanation for the observed association between neighborhood-level COVID-19 death rate and the increase in neighborhood-level drug overdose mortality rates in NYC, as both epidemics shared similar risk factors at the neighborhood level (Zhong et al. 2022). Moreover, the greater increases in overdose and COVID-19 death rates in some of the same neighborhoods suggest an unequal distribution of the burden of deaths. Unfortunately, many of the neighborhoods experiencing higher overdose fatalities were also those with documented social inequities in NYC, particularly in public health. (Kang et al. 2020).

Another possible explanation for the association between COVID-19 death rates and drug overdose fatalities during the pandemic is that individuals experiencing loss and bereavement due to COVID-19 may turn to drugs and opioids to cope with pain, depression, and traumatic grief. Previous research has documented this link between coping mechanisms (e.g., stress, traumatic grief etc.) and drug overdose (Abramson 2021; MacLean et al. 2019). Therefore, it is particularly plausible that given the limited social interactions during the pandemic, which significantly reduced activities such as visiting sick relatives and friends,  individuals could have resorted to drug use as a substitute to numb their pain and cope with their grief. The sense of loneliness brought on as a result of limited social interactions could also have played a role in individuals turning to drugs to cope. In fact, recent research has shown that social distancing was a risk factor for loneliness which in turn increased the risk of mental health conditions (Ernst et al. 2022). Additionally, the guilt and distress associated with being unable to be with loved ones before their passing due to COVID-19, as well as restrictions on performing desired funeral rites, may have driven some individuals further to excessive drug use as a means of coping with the aftermath of losing loved ones. Unfortunately, engaging in this behavior could quickly have become fatal, especially in the presence of other comorbidities.

Furthermore, restricted access to grief counseling and support as well as familial support during the pandemic (Hosseinzadeh et al. 2022) may have influenced individuals to turn to drugs, even if they had not previously been using. Similarly, reduced health services during the pandemic may have influenced individuals to turn to drugs. Individuals with chronic illnesses in severe pain may have turned to drugs to manage their pain due to reduced health service or inability to get their medications. Reduced mental health services and untreated mental health issues, which were already on the rise in NYC before the pandemic (Hamwey et al. 2024; Penninx et al. 2022), likely contributed to this trend as well (Tomko et al. 2022). These negative psychological and physical consequences of COVID-19 deaths could also have led to suicidal thoughts in individuals already at risk for fatal drug overdose. Additionally, the financial hardships resulting from COVID-19, particularly due to deaths, may have further exacerbated drug overdose rates in communities already facing socioeconomic inequality (Roman et al. 2022; Mutikani 2020).

This study has several limitations. Due to the lack of access to individual-level data, we were unable to examine the effects of covariates such as age and race or conduct subgroup analyses. Additionally, the absence of monthly overdose data limited our ability to perform more detailed temporal analyses. In 2021, the Office of the Chief Medical Examiner introduced a more robust toxicology testing for xylazine, which may have contributed to the observed increase in drug overdose deaths. On the other hand, borough-level data excluded nonresidents, potentially resulting in underreporting the total number of overdose deaths in NYC (New York City Department of Health and Mental Hygiene 2023c). It is plausible that the increase in overdose mortality associated with the COVID-19 pandemic might be due to reduced access to medications for opioid use disorder, increased isolation and loneliness, and other mediating factors. Due to the lack of contextual data, we were unable to explore the mechanisms underlying the association between COVID-19 mortality and increase in drug overdose mortality.

Our study offers some benefits, including heightened awareness of the neighborhood-level impact of COVID-19 mortality and potential future pandemic on drug overdose mortality in NYC. It also helps identify priority areas for strengthening NYC’s health systems and resilience effort concerning drug overdose. As public health practices, policies, and overdose prevention programs are developed to address the drug overdose epidemic in NYC, the influence of pandemic-related deaths at neighborhood level should be considered an important driver of drug overdose mortality (Metzl et al. 2020).

## Conclusions

In conclusion, our study found a significant correlation between COVID-19 death rates and the increase in drug overdose mortality rates in NYC neighborhoods during the COVID-19 pandemic. Neighborhoods with elevated COVID-19 death rates experienced a greater increase in drug overdose mortality from pre-COVID (2019) to during-COVID (2020–2021). Although the pandemic has been managed, the impact of COVID-19 deaths on overdose mortality during its peak highlights the potential risk a future pandemic could pose. This underscores the need for better preventive measures to address existing mental health needs and for robust programs to mitigate the mental health stress caused by pandemics.

## Data Availability

The drug overdose mortality data is not publicly available. We obtained the data from NYC DOHMH. However, the NYC COVID deaths data is available on GitHub repository and can be accessed at: https://github.com/nychealth/coronavirus-data/tree/master/totals.

## Abbreviations

ACS: American Community Survey
CI: Confidence Interval
COVID-19: Coronavirus disease 2019
DOHMH: Department of Health and Mental Hygiene
NCHS: National Center for Health Statistics
NYC: New York City
SD: Standard Deviation
UHF: United Hospital Fund
